# Structural Evolution during Chemical and Electrochemical Intercalation Reactions Probed with X-rays, Neutrons, and RF Pulses

**DOI:** 10.1063/4.0000910

**Published:** 2025-10-27

**Authors:** Sarah Ko, Kent J. Griffith

**Affiliations:** UC San Diego, San Diego, CA, USA

## Abstract

Electrochemical energy storage is an enabling technology for personal and industrial electronics, adoption of intermittent renewable energy, and the electrification of transportation. From a fundamental solid-state chemistry perspective, and in the context of batteries, it is interesting to explore new mixed ionic–electronic conductors that can withstand large changes in composition and electronic configuration over ∼1000 charge– discharge cycles to function as electrode materials and to explore new pure ion conductors with extremely low electronic conductivities that could function as solid electrolytes or interfacial coatings. Understanding the mechanisms that facilitate ion and/or electron transport or induce material degradation are the keys to discovering and engineering the next generation of battery materials.

Relatively few unique crystal structures underpin most battery materials. We are particularly interested in novel complex oxides that might offer new insights into structure–property relationships or even new performance characteristics. This talk will focus on recent examples from our lab: (i) defects, electrochemistry, and metal–metal bonding in NaNb_7_O_18_ and NaNb_13_O_33_ framework structures (Wadsley–Roth derivatives) with tunnel-blocking defects, and (ii) new lithium-rich “layered” structures, Li_3_*M*O_4_ (*M* = Nb, Ta) synthesized via instantaneous ion exchange in a molten salt flux. Both families of materials are characterized with an ‘NMR crystallography’ approach that combines X-ray and neutron diffraction with DFT-supported solid-state NMR spectroscopy.

